# Applying the DSM-5 Alternative Model of Personality Disorders and the Shedler-Westen Assessment Procedure to the Classic Case of “Madeline G.”: Novice and Expert Rater Convergences and Divergence

**DOI:** 10.3389/fpsyg.2022.794616

**Published:** 2022-02-15

**Authors:** Alisa R. Garner, Natalie Blocher, David Tierney, Megan Baumgardner, Alayna Watson, Gloria Romero, Rebecca Skadberg, Taylor Younginer, Mark H. Waugh

**Affiliations:** ^1^Department of Psychology, University of Tennessee-Knoxville, Knoxville, TN, United States; ^2^Oak Ridge National Laboratory, Oak Ridge, TN, United States

**Keywords:** AMPD, level of personality functioning, SWAP, “Madeline G.”, clinical ratings

## Abstract

Prior research supports the learnability of the *Diagnostic and Statistical Manual of Mental Disorders, 5th Edition* Alternative Model of Personality Disorders (AMPD). However, researchers have yet to compare novice ratings on the AMPD’s Level of Personality Functioning Scale and the 25 pathological personality traits with expert ratings. Furthermore, the AMPD has yet to be examined with the idiographic Shedler-Westen Assessment Procedure (SWAP). We compared the aggregated AMPD clinical profile of a group of psychology doctoral students who learned the AMPD to high levels of reliability to that of an expert rater using the crucible of the classical case of “Madeline G.” Examination of AMPD and SWAP ratings of “Madeline G.” revealed excellent overall concordance but suggests that novice raters tend to perceive lower levels of personality impairment.

## Introduction

The categorical model of psychiatric classification, including personality disorders, is increasingly criticized (Widiger and Samuel, 2005; Widiger and Trull, 2007; Krueger et al., 2014; Krueger and Hobbs, 2020). The categorical diagnostic paradigm is incorporated within the various editions of and up to the *Diagnostic and Statistical Manual of Mental Disorders, 5th Edition* (DSM-5; American Psychiatric Association [APA], 2013). While a dimensional approach was attempted but not achieved in the DSM-5 (Zachar et al., 2016), a hybrid categorical-dimensional approach termed the Alternative Model for Personality Disorders (AMPD) was placed in Section III as an Emerging Model and Measure.

The AMPD incorporates a pan-theoretical approach to personality assessment (Pincus, 2011). It includes Criterion A and B, both of which draw from important historical traditions in personality and psychopathology (Skodol et al., 2015; Waugh et al., 2017). Criterion A assesses impairment with the Level of Personality Functioning Scale (LPFS), which spans the two domains of self-functioning (i.e., Identity and Self-Direction) and interpersonal functioning (i.e., Empathy and Intimacy). Criterion A draws heavily on psychodynamic, interpersonal, and narrative psychological traditions (Bender et al., 2011), and a positive presence of personality disorder by the LPFS is the threshold for applying Criterion B. Criterion B includes 5 broad pathological personality domains, which can be further specified through 25 traits. The five maladaptive personality domains (i.e., negative affectivity, detachment, antagonism, disinhibition, and psychoticism) correspond to the Five-Factor Model, which represents the multivariate paradigm of personality assessment (Wiggins, 2003; Mulay et al., 2018; Widiger and McCabe, 2020). Personality disorder diagnosis with the AMPD is operationalized by conjoint application of impairment in personality functioning as well as pathological personality trait specifications.

A subject of interest includes the learnability of the AMPD. A recent review of AMPD research consistently showed acceptable interrater reliability across studies (Zimmerman et al., 2019). For example, a pilot study by Zimmermann et al. (2014) examined the learnability of the AMPD among 22 untrained undergraduates and found an individual intraclass correlation coefficient (ICC) of 0.55, with an aggregated rater ICC of 0.96. Another study involving 13 psychology doctoral students found an aggregated ICC of 0.98 for Criterion A and an average aggregated ICC score of 0.90 for the 5 Criterion B domains (Garcia et al., 2018). These studies demonstrate the learnability of the AMPD among untrained undergraduates and novice raters (i.e., graduate students).

Prior research showed, in general, clinical judgment and accuracy increases with experience (Eells et al., 2005; Spengler et al., 2009; Spengler and Pilipis, 2015). Zimmermann et al. (2014) found that untrained undergraduates’ total LPFS scores were significantly correlated with expert raters; however, there was a tendency to underestimate impairment in personality functioning, and reliability was relatively poor for the Empathy subscale. Similarly, Roche et al. (2018) noted a tendency to under-report total LPFS scores by research assistants compared to expert ratings, though the study does not specify the level of education or training for the research assistants, with rater reliability of the Empathy scale being the lowest. These studies are limited in (1) their use of untrained undergraduates as the comparison group as opposed to graduate students in training, and (2) their lack of specification in rater training. Few et al. (2013) expanded upon research on the AMPD by examining Criterion B ratings among novice raters (i.e., graduate students). Their findings suggest that clinicians in training may have difficulty detecting certain personality traits, including emotional lability and perseveration. However, Few et al. (2013) did not compare novice raters’ Criterion B scores with expert raters. No prior research examined novice ratings on the AMPD’s LPFS and 25 personality traits (Criterion B) in comparison to expert raters.

Further examination of the AMPD showed LPFS and Criterion B routinely reach appropriate levels of validity and internal consistency across studies (Morey and Hopwood, 2013; Zimmerman et al., 2019). Moreover, emerging research suggests convergence between the AMPD’s Criterion B with another major personality inventory (Anderson et al., 2013), the PSY-5 scale of the Minnesota Multiphasic Personality Inventory-2 (MMPI-2, Graham, 1993). Nonetheless, researchers have yet to examine the concordance between the AMPD and the Shedler-Westen Assessment Procedure (SWAP; Shedler and Westen, 2007).

The SWAP (Shedler and Westen, 2007) uses a Q-Sort methodology administered by a clinician to quantify features of personality psychopathology (Westen and Shedler, 1999a,b). The assessment makes use of 200 personality descriptions sorted into eight separate categories in terms of their descriptiveness with a fixed score distribution (Blagov et al., 2012). The measure yields various normative results, including locating a patient within a dimensional taxonomy of personality psychopathology (Westen et al., 2012). The SWAP consistently demonstrates high inter-rater reliability (Westen and Muderrisoglu, 2003; Marin-Avallen et al., 2005), even among novice raters (Marmarosh et al., 2010). Emerging research demonstrates the utility of the SWAP in distinguishing personality styles among raters (Marmarosh et al., 2010).

Both the AMPD and the SWAP make use of clinical ratings, provide dimensional metrics, and can yield results for categorical personality diagnosis. Importantly, the AMPD at its core is a dimensional model, while the SWAP derives from a prototype model in which a patient is described in terms of degrees of similarity to an idealized syndrome. Unlike the AMPD, the SWAP scales were not derived from the Five-Factor Model (Shedler, 2015; Widiger and McCabe, 2020). However, empirical overlap exists between clinicians’ ratings on the SWAP and the Five-Factor Model (Mullins-Sweatt and Widiger, 2007; Tanzilli et al., 2018). Given this similarity, it stands to reason the SWAP and AMPD would demonstrate a high level of concordance in the assessment of a patient. No prior study examined the concordance between the AMPD and SWAP personality profiles. To address the aforementioned gaps in the literature, we compared ratings among a group of psychology doctoral students to an expert rater on the AMPD LPFS and Criterion B and examined the concordance in clinical profiles between the AMPD and SWAP with the case of “Madeline G.”

## Materials and Methods

### Participants

The novice raters were 17 clinical or counseling psychology doctoral students at a large, Southeastern United States university in an advanced psychometrics course. All novice raters completed graduate coursework and practicum training in psychological assessment prior to the psychometrics course. The expert rater was a clinical psychologist with 40 years of clinical experience, significant expertise with the AMPD, and substantial familiarity with the rating target. This included co-editing a book on the re-assessment of “Madeline G.,” co-authoring a chapter on her psychodynamic assessment, and multiple professional presentations on the assessment of the subject (Hopwood and Waugh, 2020).

### “Madeline G.”

“Madeline G.” is a Native American Indian woman who participated in a comprehensive series of personality assessments spanning two decades. In 1999–2000, she completed an initial personality assessment by exemplar clinicians using different paradigms, including psychodynamic, interpersonal, personological, multivariate, and empirical (Wiggins, 2003). In 2017, Madeline agreed to a re-assessment, again by expert clinicians of different assessment paradigms, which was organized and delivered within a Therapeutic Assessment framework (Finn, 2020; see Hopwood and Waugh, 2020 for full history and assessment results and interpretations). Assessment data were drawn from many instruments, including the Rorschach Inkblot Test using the Performance Assessment System (Meyer et al., 2011), Thematic Apperception Test (Murray, 1943), Object Relations Inventory (Blatt et al., 1979), Washington University Sentence Completion Test (Hy and Loevinger, 1996), NEO Personality Inventory-Revised (NEO-PI-R; Costa and McCrae, 1992), MMPI-2-Restructured Form (Ben-Porath and Tellegan, 2008), Continuous Assessment of Interpersonal Dynamics (Sadler et al., 2015), and numerous measures based on the Interpersonal Circumplex (Pincus et al., 2020).

From both Wiggins (2003) and Hopwood and Waugh (2020), Madeline’s history is noted to include significant trauma, including physical and emotional abuse, exposure to her parents’ alcohol misuse, interpersonal violence, and her mother’s suicide attempt. She spent much of her childhood in foster care, began living on her own at age 12, and served time in prison during her teenage years. While in prison, Madeline resolved to improve her life and eventually obtained advanced education and began a career as a defense attorney for underprivileged clients. Madeline was involved with her common-law husband for 7 years; however, she experienced depression with suicidal ideation and became more socially isolated after he unexpectedly left the relationship. Shortly before her reassessment 17 years later, Madeline cared for her dying uncle, the only family member with whom she felt close. This precipitated a difficult period of emotional distress and personal and professional transition. Accordingly, the collaborative assessment was organized around self-generated questions about her identity, sense of self, life direction, and future goals (Hopwood and Waugh, 2020).

### Procedure

Over 8 weeks, novice raters were trained in the AMPD and SWAP through didactic study and readings, including Section III of the DSM-5 (American Psychiatric Association [APA], 2013). Training in the AMPD included practice ratings of multiple clinical vignettes and comparison to expert criterion ratings. Novice raters studied the case of “Madeline G.” through reading the entirety of *Personality Assessment Paradigms and Methods: A Collaborative Reassessment of Madeline G.* (Hopwood and Waugh, 2020), that included Madeline’s narratives, test data, and interpretations from expert assessors skilled in different assessment paradigms. Novice raters then provided their anonymous scores of “Madeline G.” on the AMPD and SWAP. For the AMPD, ratings were averaged and rounded to obtain the novice raters’ averaged clinical profile. Ratings on the SWAP descriptions were averaged and then organized in descending order. The eight highest averaged descriptions were assigned the highest value on the SWAP (i.e., a “7” or “most descriptive”). The next 10 highest averaged descriptions were assigned the second highest value (i.e., a “6”). This process continued for the remaining set of values until all descriptions received a rating. Items for which multiple ratings of the same average score that were assigned to different SWAP values (e.g., for two ratings of 3.55, one had to be assigned a 3 and one a 4) were discussed among raters to determine the appropriate value. The novice raters’ averaged clinical profiles were then compared to the ratings of the expert rater.

### Measures

*Alternative Model of Personality Disorders* (AMPD; American Psychiatric Association [APA], 2013). The AMPD is a hybrid categorical-dimensional approach to personality disorder diagnosis consisting of the LPFS (Criterion A) and Criterion B. The LPFS assesses impairment in personality functioning, including identity, self-direction, empathy, and intimacy. Ratings on each of the four areas are provided on a 5-point Likert scale ranging from 0 (*little or no impairment*) to 4 (*extreme impairment*). The LPFS threshold for personality disorder is met when two or more areas of personality functioning are rated at two (*moderate impairment*) or higher. Criterion B includes pathological personality domains and traits within those domains. For the present study, ratings were provided for the traits utilizing the Personality Disorder Level-Trait Clinician Rating Form (Waugh, 2014; Garcia et al., 2018). Ratings for each personality trait were provided on a 4-point Likert scale ranging from 0 (*very/often false*) to 3 (*very/often true*), with a score of two or more indicating the presence of the pathological personality trait. The AMPD demonstrated reliability and validity as an assessment of personality disorder (Garcia et al., 2018; Zimmerman et al., 2019).

*Shedler-Westen Assessment Procedure* (SWAP; Shedler and Westen, 2007). The SWAP is an idiographic personality assessment procedure which utilizes the clinician’s judgment to provide personality and diagnostic profiles. The SWAP contains 200 descriptive statements which the clinician sorts into eight categories ranging from 0 (*not at all descriptive of patient*) to 7 (*most descriptive of patient*). The SWAP utilizes a Q-sort method where a fixed number of statements are organized into the eight categories. The sorted statements are then converted to personality trait scales containing *T* scores (*M* = 50, *SD* = 10). *T* scores of greater than 55 suggest clinical elevations on the respective personality trait scale with the exception of the Psychological Health Index, a global measure of ego and interpersonal strengths, where *T* scores of greater than 50 indicate greater psychological resources and capacities (Shedler and Westen, 2007). Prior research supports the general reliability and clinical validity of the SWAP (Bradley et al., 2007; Shedler and Westen, 2007; Smith et al., 2008; Blagov et al., 2012; Smits et al., 2021).

### Data Analytic Strategy

Two-way, random effects model absolute agreement ICC of the 17 novice raters’ scores on the AMPD (LPFS plus Criterion B traits) and SWAP (200 descriptive statements), both single and average, were conducted using SPSS version 25. Next, the ICCs were calculated for the novices’ averaged ratings and the expert’s ratings on the AMPD and SWAP to show novice raters’ agreement with the expert rater. Qualitative descriptors for the ICC were as follows: poor ≤ 0.40, fair = 0.41–0.59, good = 0.60–0.74, and excellent ≥ 0.75 (Cicchetti, 1994). We then compared the novice raters’ averaged AMPD and SWAP ratings to the expert’s ratings. We considered differences meaningful when expert and novice ratings yielded discrepancies in meeting a clinical threshold (score of 2 for AMPD, 55 for the SWAP, 50 for the SWAP’s Psychological Health Index). Novice and expert rater clinical interpretations, derived from the AMPD and SWAP, were then compared.

## Results

See Table 1 for ICC results. Novice raters showed good and excellent interrater reliability, respectively, for the full AMPD, single rater ICC [2, 1] = 0.73 and average ICC [2, 16] = 0.98. Interrater reliability for the SWAP was fair for single rater, ICC [2, 1] = 0.55, and excellent for average, ICC [2, 17] = 0.96. ICCs were then examined to compare novice raters’ averaged AMPD and SWAP ratings to the expert rater. Results of the two-way, random effects model ICC showed fair and good agreement, respectively, for the full AMPD, single rater ICC [2, 1] = 0.58 and average ICC [2, 2] = 0.74. Agreement for the SWAP was excellent for both single rater, ICC [2, 1] = 0.78, and average, ICC [2, 2] = 0.89.

**TABLE 1:** Intraclass correlations (ICC) of the raters on the Alternative Model of Personality Disorders (AMPD) and Shedler-Westen Assessment Procedure (SWAP).

	AMPD	SWAP
**ICC of novice raters**		
Single	0.73	0.55
Average	0.98	0.96
**ICC of novices’ averaged ratings and expert rater**		
Single	0.58	0.78
Average	0.74	0.89

**FIGURE 1:**
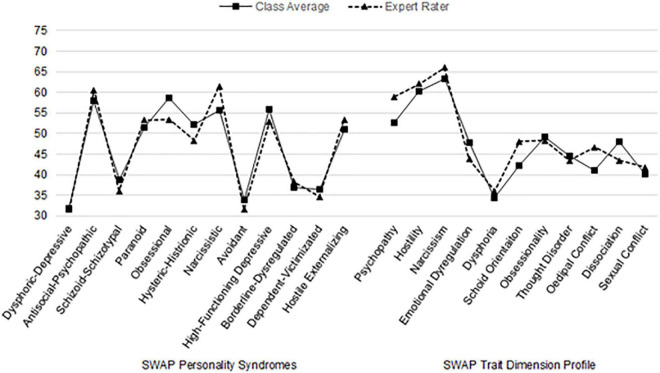
Comparison of graduate students’ and expert’s Alternative Model of Personality Disorders (AMPD) clinical ratings of “Madeline G.” Criterion A scored on a scale of 0–4. Criterion B scored on a scale of 0–3. Scores of 2 or higher indicate impairment or presence of personality facet (American Psychiatric Association [APA], 2013; Waugh, 2014; Garcia et al., 2018).

For the AMPD, aggregated LPFS rating results showed both novice and expert raters noted personality impairments consistent with a diagnosis of a personality disorder. Novice raters found moderate levels of impairment in areas of personality functioning related to identity (*M* = 1.5, rounded to 2) and intimacy (*M* = 1.8, rounded to 2), while the expert rater noted additional impairment in empathy (see Figure 1 for AMPD results). Novice raters noted 6 areas of elevation on Criterion B maladaptive personality traits, while the expert rater noted 12. Novice raters’ Criterion B ratings on the AMPD were lower than the expert ratings and did not exceed clinical thresholds in areas of callousness, deceitfulness, manipulativeness, perseveration, risk taking, separation insecurity, and suspiciousness. On the other hand, novice raters evaluated Madeline as showing greater intimacy avoidance than the expert rater.

**FIGURE 2:**
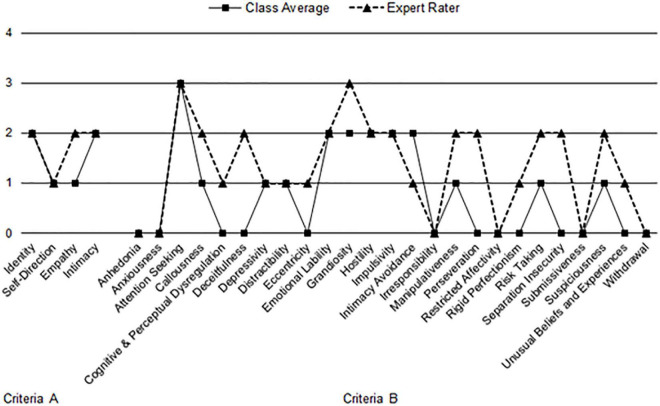
Comparison of graduate students’ and expert’s Shedler-Westen Assessment Procedure (SWAP) clinical profile of “Madeline G.” *T* scores of 55 or greater indicate clinical elevation (Shedler and Westen, 2007).

For the SWAP, both novice raters (*T* = 63.7) and the expert rater (*T* = 58.8) viewed Madeline as showing a high level of overall psychological health (see Figure 2 for SWAP results). Both novice and the expert rater yielded T scores > 55 for antisocial and narcissistic personality syndromes, and for hostility and narcissistic traits. The novice raters rated Madeline significant for obsessional and high-functioning depressive, whereas the expert rater’s SWAP-200 profile was sub-threshold for these qualities. The expert rating for the psychopathy personality trait met clinical threshold, while novice ratings were below.

Clinical profiles on the AMPD and SWAP among novice raters and expert rater showed similarities. Novice raters’ profiles suggested someone with a level of impairment in identity and intimacy consistent with a personality disorder but who also possesses relatively robust ego strength and points of emotional strength serving as protective factors. Novice raters’ profiles characterized Madeline as a woman who is highly attention seeking. She finds meaning in challenging “the system” and helping the underdog but shows deficits in intimacy functioning. Emotionally she is labile, hostile, and impulsive. She possesses a grandiose sense of self, expecting herself to be “perfect” and invulnerable. She has a high need to be in control of situations, which results in conflict with others, especially with authority figures. She views her shortcomings as related to external factors rather than reflecting on her own role in problems. The expert rater’s profile noted impairment in identity, intimacy, and empathy consistent with a personality disorder but suggested that she demonstrates pockets of ego strength and resilience. The expert rater’s profile also suggested Madeline has a grandiose sense of self with an exaggerated sense of self-importance expressed in attention seeking and a need to hide her true, vulnerable self from others. She can be deceitful, manipulative, and is prone to take risks. Emotionally, she is callous, labile, hostile, and impulsive. Toward others she is suspicious but fears being alone or rejected. She will continue engaging in a way of doing things well after it has been shown to be ineffective.

## Discussion

Our findings showed strong interrater reliability among the novice raters for the AMPD, but only fair to good agreement with the expert rater. While both the aggregated novices’ and expert’s ratings detected the presence of a personality disorder on the LPFS, the novice raters did not detect the moderate level of difficulty in empathy noted by the expert rater. Similarly, the expert rater’s Criterion B ratings showed a greater number of elevations compared to novice raters. Notable differences included callousness, deceitfulness, manipulativeness, perseveration, separation insecurity, and suspiciousness.

On the SWAP, novice raters had excellent agreement with the expert rater, suggesting better agreement on the SWAP compared to the AMPD. Differences in level of agreement between measures may reflect methodological distinctions. The AMPD requires raters to assign values based on a Likert scale; raters can immediately assign clinical elevations with the rating of a 2, while the SWAP has raters sort descriptive statements in which it may be unclear at face value how sorting will affect clinical elevations. The SWAP’s Q-sort methodology is designed to reduce rater error variance (Westen and Shedler, 2007). It is possible that novice ratings aligned more with the expert in the SWAP due to the ambiguous nature of the assessment and discomfort assigning clinical elevations on the AMPD. It is also true that the greater agreement on the SWAP is partly because the SWAP contains 200 items, while the full model AMPD contains 29, and it is known that psychometric reliability increases with a greater number of items. Comparison of the novice raters’ vs. expert rater’s SWAP profiles also show a similar pattern in personality traits, but with decreased elevations on the SWAP profile among the novice raters compared to the expert rater. This is consistent with the AMPD profile comparison that novice raters may have difficulty detecting the presence and severity of certain personality traits.

Both the novice raters and the expert rated Madeline’s psychological health index on the SWAP as high. While this may seem counter to the ratings on the LPFS, these scores assess two different constructs. While the LPFS pertains to impaired functioning related to the self and others, the SWAP’s psychological health index is broader in scope and assesses both psychological impairment and coping resources. Importantly, the psychological health index on the SWAP includes items pertaining to one’s tendency to be assertive, articulate, energetic, outgoing, to enjoy challenges, and derive a sense of pleasure from accomplishment. Psychological resources such as these likely contribute to Madeline’s success in her career and explain why the psychological health index of the SWAP was elevated across raters despite the LPFS ratings, which suggest significant dysfunction.

Examination of the AMPD and SWAP results suggested concordance in the clinical interpretations of these two profiles. Novice raters were consistent in noting problems in intimacy, attention seeking, hostility, impulsivity, and grandiosity. The expert rater was consistent in noting difficulties in intimacy and empathy (e.g., deceitfulness and manipulativeness), hostility, impulsivity, risk-taking, and fears of rejection.

Our results also replicate previous study findings, which suggest overall agreement on the LPFS between novice raters and expert raters (Zimmermann et al., 2014; Garcia et al., 2018). Notably, this study and prior studies suggest the empathy domain of the LPFS can be especially problematic for raters lacking experience (Zimmermann et al., 2014; Roche et al., 2018). Roche et al. (2018) observed that research assistants’ ratings on the LPFS domain of empathy alone did not correlate with self-report ratings on the LPFS, including the self-report empathy rating. They suggested that life history interviews (which they utilized) may more easily evoke information about the other three LPFS domains (i.e., identity, self-direction, and intimacy). Similarly, Zimmermann et al. (2014) recommended that the finding of lower reliability in performing empathy ratings suggests the importance of providing rater training on how to inquire for and infer manifestations of impaired empathy. Application of the LPFS in general involves a higher level of inference than for the maladaptive traits of Criterion B (see Mulay et al., 2018), and inspection of the LPFS domains as presented in the *DSM-5* suggests that empathy is relatively less behavioristic than identity, self-direction, or intimacy functioning. This may result in less consistency in clinical ratings.

Our study also extends upon past research by examining overall agreement between novice and expert raters on Criterion B. Our findings suggest there appear to be some notable differences in novice raters’ abilities to rate some Criterion B personality traits. This highlights how differences in training and clinical experience may impact the formulation of diagnostic impressions and the ability to assess more complex facets of personality traits and functioning. Extant literature similarly suggests that novice raters generally exhibit differences in their ability to conceptualize clinical cases (Eells et al., 2005) and accurately diagnose (Lambert and Wertheimer, 1988) when compared to more expert raters. This recurring finding suggests the importance of adequate training in personality assessment and its subtleties. Early introduction to concepts embedded within the AMPD may increase students’ sensitivity to how psychopathology can be expressed.

The present study has some limitations. As it involved analysis of one case, results should be interpreted as a demonstration case and may not be generalizable to other cases. Furthermore, rater review of the assessment materials included results and interpretation of the NEO-PI-R scores for Madeline, which could confound novices’ ratings on Criterion B of the AMPD, as it shows convergence with five factor models. Despite these limitations, a very significant amount of case history, raw test data, and the case description relied upon multiple interviews and assessments. All novice raters were involved in training programs at the same university, which possibly influenced the level of concordance. Furthermore, the current study only utilized one expert rater due to limitations in resources and availability of those with substantial familiarity with the entirety of the case of “Madeline G.” It would be beneficial to replicate this study among a larger, more diverse population of novice and expert raters. Additionally, the use of clinical vignettes/testing materials does not always replicate assessments based upon face-to-face contact. However, use of standardized case material is a regular component of training programs.

Our findings underscore the need for training that focuses on comprehension and assessment of personality functioning and traits. Our findings combined with past similar findings highlight the necessity of thorough training in the AMPD and SWAP that includes comparison and conference of novice and criterion ratings. This could be achieved through repeated exposure to and practice ratings of case vignettes. Garcia et al. (2018) noted significant improvements in interrater reliability, especially for the empathy subscale, after three rounds of training. Training programs should also emphasize the importance of utilizing multiple assessments to ensure trainees obtain the information needed to best assess impairments in functioning and personality. Future research should examine potential discordance in the perception of personality pathology among novice and expert raters. Additional studies would benefit from examining differences in the AMPD and SWAP among groups that are of commensurate size, as well as the presence of within-group differences among individual raters (e.g., gender, theoretical orientation). Further, these results provide evidence for the concordance between the overall clinical interpretations of the AMPD and SWAP; thus, clinicians should consider whether the use of both methods provides incremental understanding to the conceptualization of their clients.

## Data Availability Statement

The raw data supporting the conclusions of this article will be made available by the authors, without undue reservation.

## Ethics Statement

Ethical review and approval was not required for the study on human participants in accordance with the local legislation and institutional requirements. Written informed consent for participation was not required for this study in accordance with the national legislation and the institutional requirements.

## Author Contributions

AG and MW conceived the study idea and study design, initiated the study, contributed to writing the manuscript, and coordinated and finalized the article. NB, DT, MB, AW, GR, RS, and TY provided input on the study design and contributed to writing the manuscript. All authors read the manuscript, provided feedback, and approved the final version.

## Conflict of Interest

The authors declare that the research was conducted in the absence of any commercial or financial relationships that could be construed as a potential conflict of interest.

## Publisher’s Note

All claims expressed in this article are solely those of the authors and do not necessarily represent those of their affiliated organizations, or those of the publisher, the editors and the reviewers. Any product that may be evaluated in this article, or claim that may be made by its manufacturer, is not guaranteed or endorsed by the publisher.
